# Commentary: Idiopathic Intracranial Hypertension Without Papilledema (IIHWOP) in Chronic Refractory Headache

**DOI:** 10.3389/fneur.2019.00039

**Published:** 2019-01-29

**Authors:** Roberto De Simone, Angelo Ranieri

**Affiliations:** ^1^Department of Neuroscience, Reproductive Sciences and Odontostomatology, Headache Centre, University Federico II of Naples, Naples, Italy; ^2^Division of Neurology and Stroke Unit, Hospital A. Cardarelli, Naples, Italy

**Keywords:** idiopathic intracranial hypertension without papilledema, chronic migraine, lumbar puncture, pathogenesis, sinus stenosis

We read with interest the paper Idiopatic Intracranial Hypertension without papilledema in Chronic Headache by Favoni et al. (1) and wish to congratulate with the Authors. Actually, their data independently confirm our original main findings (2) that a raised intracranial pressure may be causatively involved the progression of migraine pain and that a single lumbar puncture (LP) with cerebrospinal fluid withdrawal may provide a long term benefit in many such patients. Moreover, their observations also substantiate our criticism to the revised IIH/IIHWOP diagnostic criteria by Friedman (3), demonstrating their unacceptably low sensitivity.

In their series of retrospectively assessed refractory CM patients they have found a 22.5% prevalence of cases with opening pressure > 200, most of which (77.8%) showed a prompt and sustained remission of chronic pain after LP. This finding is higher than IIHWOP prevalence reported in non-selected series of CM, ranging between 10 and 14% (4, 5). However, as the authors highlight, it is significantly lower than the prevalence reported in our original study (86.4%) (2). This discrepancy may relay on differences in patients selection leading to quite different populations. Actually, almost all their patients (92.5%) presented a medication overuse headache (MOH), a known risk factor for pain progression, while in our sample MOH prevalence was 59.1%. Moreover, in our screened population of 56 subjects with documented unresponsiveness to preventative treatments we found a prevalence of “bilateral transverse sinus (TS) stenosis/hypoplasia or at least unilateral segmental TS flow gap/aplasia at uncontrasted MRV” of 92.8%. Unfortunately, in the Favoni et al. work this data is not specified. However, in a previous very similar version of the paper by Favoni et al. (6) the overall sinus stenosis prevalence was only 48%.

Last, neither in the first nor in the actual version of the Favoni et al. works it is clear weather refractoriness was assessed prospectively or retrospectively. Our study was conducted on a series of CM patients with a prospectively assessed refractoriness to medical treatments, after the failure of 2 subsequent pharmacological treatments of at least 2 month each (median 12.2 weeks; range 8.3–23.8), with appropriate drugs (included in a list of 7) at adequate dosages. This procedure allowed us to select only 56 out of 278 patient (20.1%) consecutively diagnosed with CM at our tertiary headache center. Again, in both versions of the Favoni et al. works (1, 6) the size of the original screened population of CM patients is not provided.

The assessment of refractoriness is a crucial issue in headache research. CM patient referring to a tertiary headache center for the first time very often complain of a long history of disease and usually report a number of previous failed treatments and/or medication overuse withdrawal. However, this is not enough to classify them as true refractory since retrospective assessment of CM refractoriness is unrelialable (7).

## Author Contributions

RDS drafted the manuscript. All authors contributed to its revision and approved the submitted version.

### Conflict of Interest Statement

The authors declare that the research was conducted in the absence of any commercial or financial relationships that could be construed as a potential conflict of interest.
